# Trichiniasis—Nine Cases

**Published:** 1888-01

**Authors:** G. W. Furey

**Affiliations:** Sunbury, Pa.


### TRICHINIASIS—NINE CASES.

BY G. W. FUREY, M. D., SUNBURY, PA.

It has been my lot to attend this, the only case, or group of cases, of this peculiar, tedious and dangerous disease, ever known to have occurred in Central Pennsylvania.

Owing to the fact that I was entirely mistaken in my diagnosis of the first stage of the first case presented, and that this disease fortunately *is* a very rare one, though liable in the practice of any physician to confront him with its mysterious, unintelligible and apparently harmless symptoms, I will endeavor to report my recent experience, as well as my memory and the few notes I was able to take, will admit.

I shall discuss the disease in a general way first, and then in detail the phenomena presented to me in the study of the first five cases.

About the 9th of December, 1880, a young man, H. L. by name, aged twenty-six, car repairer, called on me for treatment for what he described, and which I believed, to be a bad cold. I prescribed the usual remedies for it, telling him not to go to work for a few days. The next day I was sent for to attend him, and also other members of the family with which he was boarding. I was at once struck with the strange similarity of symptoms between him and the others; and concluding that a "bad cold" never appeared so uniformly at once in so many cases, I became impressed with the conviction that I had to deal with some unfamiliar specific affection, or the result of some ill-marked poisoned condition. A search of the premises, an investigation into the manner of living, condition of the heating apparatus, cellar, out-houses, etc., soon restricted my suspicion to something which they had eaten. The peculiarity of muscular "lameness," and the unanimity with which they were complaining of it, suggested *Trichiniasis.* I asked to see the pork they were eating; was shown the barrel, and on examining some of its contents was at once struck with its singular *leanness*, and peculiar pale, un-pork-like color, and general appearance. They told me that they had bought the hog of a butcher, dressed; that it weighed three hundred and seventeen pounds; had frame enough for four hundred and fifty pounds; that it might have been a "native" or a "western" hog.

I put the five patients on the treatment which I will mention, and requested them not to eat any more of the pork until told to do so. I removed several small pieces of the meat, and called upon Professor G. G. Groff, M. D., of Lewisburg University, for the use of his microscope, and for his assistance. After careful search, we were rewarded by finding in abundance—five in one field—*Trichina Spiralis.*

The five cases collectively presented the characteristics stated by writers upon the subject, though individually they did not, with the exception of the first case I saw. In all there was early languor, œdema of the face and eyelids, hoarseness and irritable fauces; while in only two was there any noticeable swelling of the extremities. Only one was troubled with profuse perspiration; but one had gastric trouble ; one was distressed for a few days with colic, neuralgia and diarrhœa ; two had considerable bronchial trouble, all had more or less congestion of the conjunctivæ, with corresponding lameness of the orbital muscles. The febrile stage—present in all the cases—also varied in degrees and duration. Only one approached anything like delirium (H. L.), and he presented a very marked degree of disturbance of muscular and nervous co-ordination. The cephalalgia varied somewhat as to situation ; being in the anterior portion of the head in all, while two in addition had very severe pain in the posterior part. My attention was drawn to the constant presence of a special lameness of trapezius muscle. Next to be complained of were the deltoid and brachialis, and those of the lower extremities. All had difficulty of deglutition.

I noticed in the morning a marked intermission of fever, but especially of the soreness, with a want of appetite. The two specially troubled with bronchitis, were also specially sore in the region of the inter-costal muscles and diaphragm. The distress, too, corresponded with the movements of the body, and direct pressure on the muscles. The case of I. W. suggested to me the belief that the intensity of the disease depended considerably upon the amount of parasites ingested, he being under dietetic restrictions for indigestion, and in consequence had eaten sparingly of the infected pork. Another reason for these cases being so mild, according to this same idea, is that the family had been eating of a quarter of beef, and had only indulged in a few dinners of the *boiled pieces* of the pork. That they had eaten of the large pieces will account for the non destruction of parasities lying deep down in the muscles, and therefore my five cases of trichinosis, which would never have occurred if the meat had all been sliced thin and well cooked. I have been impressed more than by anything else, with the conviction that it requires either a very considerable previous experience with the disease, or a *group of cases and a microscope*, to establish a diagnosis.

When we consider that a man may have trichinosis and not be confined to bed; not "sweat profusely," not "cough extensively," nor even suffer from "nausea," nor "diarrhœa," the inefficacy of book descriptions, in one's first case at least, is very apparent. I mean that all the works I have read on the subject have considered only typical cases, while here in one family, three of whom were of the same family habits and condition, I found only one typical case, while the other four were so ill-marked and moderate that I should never have suspected any one of them, alone, of having trichinosis. For further assurance that we ought to be on the alert for such contingencies, it is only necessary to say that while I have been attending my five cases, I have read in the papers of seven cases in one family, and four in another, in Franklin, Erie county, Pennsylvania, who were treated for typhoid fever until six of the first seven died, before it was ascertained that the disease was trichiniasis; and also of one, a butcher in

New York, who was taken sick with a brief illness, and underwent the most modern approved treatment for rheumatism.

I have already mentioned that of my five cases there was only one typical case; and I have pointed out the most essential points in which the others differ from it; so that by referring to the general description, and comparing them with the record I propose giving of the first and worst case it will obviate the need of reporting all.

H. L., aged 26, began to feel languid and distressed on Sunday, December 5, 1880. Concluding that he had only a cold, he resumed work as usual on Monday, Tuesday and Wednesday. Thursday morning, the 9th, he felt entirely unable to go to work. He called on me for treatment for "his cold," and I prepared some medicine for *a cold*. Yet, I was not exactly clear as to the cause of the peculiar œdema of his hands and face; which he told me had shown itself on Monday. Friday evening I was called to see him and others of the family where he boarded. It was at once apparent that even a very bad cold would not account for his symptoms. His temperature was 103⅓°, pulse 106; face much swollen; cough dry and irritable; bowels constipated. Tongue not coated. Considerable pain in anterior and posterior parts of head. A dull, indescribable pain, or lameness in the muscles of the back, arms and limbs, and considerable distress occasioned by efforts at respiration. He preferred to lie on his back. I noticed no indication of flexion of the extremities. The fever began on Wednesday evening the 8th, and continued until Sunday. The œdema mentioned, which had attained its maximum by the 12th, wholly closing both eyes, was entirely gone by the 15th. On the 12th I noticed that the dry, irritable cough brought from his throat a tenacious sputa considerably mixed with blood. The discharges from the nostrils, though not so tenacious, were also colored. This peculiar symptom also affected in the same manner one of the other patients, a brother. During the time it remained, which was about six days, he complained of dryness of the throat, and a prickling sensation. Profuse diarrhœa began on the 15th, lasting to the 21st. The sweating was first complained of on the 17th and continued till the 24th. It was worse at night There was considerable emaciation.

When I first suspected trichinosis, I ordered thorough evacuation of the alimentary canal, which was followed by large doses of quinine. Then when I found the parasite unmistakably in the pork, I began the use of as large doses of sulpho-carbolate of soda as the stomach would bear. This, with a liberal diet, and ammonia liniment externally, constituted the treatment. Forty-two days after the first attack, I considered him to be out of danger and able to resume his work.

In the face of the discouragement afforded me by the perusal of several works on the subject, Ziemssen's Cyclopœdia, for one of them, I must, with all deference and some modesty, affirm that were I to be called again to attend a similar case or group of cases, instead of pursuing the weird and shadowy pathway offered me by the authors referred to, I should, and I think conscientiously, follow my above-mentioned treatment.

The foregoing cases occurred in the fall of 1880 In the fall of 1885, a German, having supplied his larder with plenty of "city-cured hams," proceeded, as a matter of course, to slice one of them off, and eat of it raw. In a few days his family physician was called in to attend his little eight-year old daughter. In the course of treatment, consultation was had, and the child treated for rheumatism. After suffering terribly for about a week, she died. About this time the father and a little son, aged five years, began to manifest symptoms similar to those the little girl complained of at first. I was sent for, and after getting a description of the girl's case, together with all the attending circumstances, immediately pronounced the trouble to be trichiniasis. The father gave me no difficulty at all; but it was far different with the little boy. He rapidly developed all the classical symptoms and more too. I had no hopes of his recovery; but, after following the treatment I have mentioned above, with the addition of santonine, I was rewarded by a complete recovery of both my patients. I verified the diagnosis by examining the fæcal matter under a lens, the ham which caused the disease having been all eaten.

Two years ago this fall, I was called down to Bethlehem, to see the two surviving cases of that sad group of trichinosis. The mother, and thirteen-year-old daughter had recently died of it. I found the father, though not confined to the bed, suffering very much indeed, while the little five-year-old girl was lying very low. The same treatment was ordered as the above, with, of course, tonic and sustaining measures. Both recovered, and when they visited me, a year after, were enjoying usual health.

The mother and daughter, who died, were, I was informed, treated for rheumatism during the first part of their sickness, which teaches the lesson that it is necessary to be extremely particular in diagnosing rheumatism. To Mr. Eugene A. Reau, of Bethlehem, the accomplished microscopist is due the credit of first suspecting and demonstrating these cases to be trichinosis. He prepared some of the best specimens, both photographs and slides, from muscles of the patients who died, that I ever saw. No doubt he would be willing to furnish them to any person desiring them.

The facts in this report are: In the space of five years, in my practice, I have encountered three groups of this troublesome affection, in which, every time, the first cases were mistaken for something else. Three cases, associated with the nine I treated for trichiniasis, and which were treated for rheumatism, died. The nine subjected to the treatment I have mentioned, recovered completely. I do not wish to imply that it is infallible, or that I think it is; but I would certainly rely on it were I called to see other cases. I would be pleased to correspond with any physician who has had experience with the disease.—*Medical and Surgical Reporter.*

